# Sexual harassment protocols at the European universities: An overview of key components and recommendations for improvement

**DOI:** 10.1371/journal.pone.0345002

**Published:** 2026-03-20

**Authors:** Marina Berbegal-Bernabeu, Vanesa Pérez-Martínez, Mafalda Sousa, Sofia Neves, Anneleen De Cuyper, Stefano Porru, Angela Carta, Maryna Manchenko, Sylwia Jaskulska, Barbara Jankowiak, Marlies Wallner, Viktoria Stifter, Carmen Vives-Cases

**Affiliations:** 1 Department of Community Nursing, Preventive Medicine and Public Health and History of Science, University of Alicante, Alicante, Spain; 2 Department of Social and Behavioral Sciences, University of Maia, Maia, Portugal; 3 Interdisciplinary Centre for Gender Studies, University of Lisbon, Lisbon, Portugal; 4 Department of Sociology, University of Antwerp, Antwerp, Belgium; 5 Department of Diagnostics and Public Health, University of Verona, Verona, Italy; 6 Rights and Justice Unit, CESIE ETS, Palermo, Italy; 7 Faculty of Educational Studies, Adam Mickiewicz University in Poznan, Poznan, Poland; 8 Department for Health and Social Sciences, University of Applied Sciences Burgenland, Eisenstadt, Pinkafeld, Austria; 9 CIBER of Epidemiology and Public Health (CIBERESP), Instituto de Salud Carlos III, Madrid, Spain; PNG National Research Institute, PAPUA NEW GUINEA

## Abstract

Protocols against sexual harassment (SH) have been widely adopted in European universities as part of a broader structural gender approach in higher education and research institutions. However, existing literature indicates that these protocols have often been insufficient. In particular, there is a lack of effective restorative measures that address the needs of both survivors and perpetrators of SH. The aim of this study was to analyse and compare SH protocols across seven European universities and to provide evidence-based recommendations for improving the management of SH in higher education. The study adopts an intersectional perspective, recognising how overlapping social factors (such as gender, ethnicity, disability, or migration status) shape experiences of SH. This study employed a qualitative content analysis, using content analysis of 10 SH protocols from seven European universities. The results show that some universities provide comprehensive protocols that include procedural, preventive, and reparative measures. However, others have protocols with significant shortcomings. These include limited implementation, diffuse procedures, and a lack of practical application of an intersectional approach. Main recommendations include offering multiple and accesible reporting and support resources, appointing a single case coordinator to accompany survivors throughout the entire process, and enhancing cooperation between departments and specialised external services addressing SH and sexual violence (SV).

## Introduction

The European Institute of Gender Equality (EIGE) [1] defines sexual harassment (SH) as “any form of unwanted verbal, non-verbal or physical conduct of a sexual nature that occurs, with the purpose or effect of violating the dignity of a person, in particular when creating an intimidating, hostile, degrading, humiliating or offensive environment”. SH is also described as a form of gender-based violence (GBV) because it disproportionately affects women [2].

SH can range from bullying and sexist jargon to sexual abuse and rape [3] and can occur in different contexts, including the workplace. Results of the European Union Agency for Fundamental Rights (FRA) survey [4] suggest that men are perpetrators in 86% of cases of sexual harassment in the workplace. Research on GBV and its consequences in European academia [5] indicated that one of the most prevalent forms of GBV at the university was SH, where almost 1 in 3 students and staff respondents were survivors. Experiencing SH has negative emotional and behavioural consequences for survivors (e.g., depression, post-traumatic stress, eating disorders, problematic alcohol use or sleeplessness [6,7]) as well as decreased academic success and job performance [8,9]. Moreover, the severity of victimisation of SH and the quality of support received in its aftermath has been found to impact these consequences [8].

SH in the university context is characterised by power inequalities, not only in terms of sex, gender, or other sociodemographic characteristics (e.g., ethnicity, age), but also university hierarchies (e.g., professors vs. students, high-status positions vs. low-status positions). For these reasons, addressing SH requires an intersectional approach that recognizes how various forms of oppression and inequality such as racism, xenophobia, sexism and classism intersect and are experienced simultaneously, shaping social relations in complex ways [10,11]. According to this perspective, individuals who belong to historically marginalized groups—along the axes of religion, age, migration status, sexual orientation, or disability—are subject to overlapping forms of exclusion and encounter aggravated and specific forms of harassment that cannot be fully understood when each category is considered in isolation [12]. For example, previous studies have found that female, younger students and sexual minority students face higher rates of SH [13,14]. Among university staff, being from a minority ethnic group is associated with a significantly increased risk of SH victimisation [15]. Including this perspective in SH protocols can help identify risks more effectively, ensure equitable access to support services, and tailor responses to diverse needs of those affected.

European universities have adopted SH protocols as a part of structural gender equality initiatives in higher education and research institutions [16]. As defined by the UniSAFE project ([17], p. 1), a protocol for addressing GBV in higher education institutions is “a document that prescribes what will happen in case inappropriate behaviour is reported. It provides a step-by-step guide on how incidents of gender-based violence are reported, addressed and resolved in the institution”.

Although SH protocols are established in most European universities, systematic reviews on SH in higher education [5] suggest that universities have not taken sufficient actions to prevent SH. Actions have not been effective or have lacked follow-up or research-based evaluation, highlighting the need for reparative measures, bystander intervention programs and sanctions for perpetrators. In addition, university protocols against SH are heterogeneous, with widely differing definitions of SH and measures applied to cases [5]. Studies point out the need for improved counselling and communication strategies to prevent SH [18]. Key elements include education and awareness, interpersonal communication skills training, provision of support resources, specialized facilitator training, comprehensive sexual education curricula, and the use of media and technology in counselling [18].

Studies outside the European context have also examined how universities develop and implement SH protocols and policies. Research in Brazilian and Central American public universities similarly reports variability in institutional procedures, limitations in implementation, and challenges in ensuring accountability [19,20], while studies on United States campuses identify differences in policy scope and institutional compliance with federal legal requirements [21,22]. Although written protocols are widespread in higher education globally, considerable variation remains in their structure, clarity, and practical application, underscoring the need for systematic description and comparison of institutional SH protocols.

Given its negative impact, unifying university efforts to tackle SH is urgent. An in-depth examination of the main characteristics of existing SH protocols in higher education institutions is needed in order to identify their key components and shortcomings. Currently, there are no studies that analyse SH protocols at the European level. The aim of this study is to analyse and compare the SH protocols of seven European higher education institutions. The study also seeks to provide recommendations on how to manage SH in higher education and for improvement of university SH protocols.

This study is a part of the inter-institutional European project “Uni4Equity” (101094121-Uni4Equity, 2023-2026), carried out at the University of Alicante (UA, Spain), University of Palermo (UNIPA – CESIE ETS, Italy), University of Maia (UMAIA, Portugal), Adam Mickiewicz University (AMU, Poland), University of Applied Sciences Burgenland (UASB, Austria), University of Verona (UNIVR, Italy) and University of Antwerp (UANTWERP, Belgium). Its main objective is to strengthen the capacity of universities to identify, map and respond to online and offline SH in the workplace and other university environments (classrooms, digital space), with an explicit (but not exclusive) focus on social minority groups. These institutions were chosen based on their demonstrated experience and commitment to gender equality policies and the prevention of SH. The collaborative structure of the consortium facilitates comparative analysis across diverse legal, institutional and sociocultural contexts, while promoting mutual learning and the exchange of good practices within the broader European regulatory and policy framework addressing violence and harassment in higher education institutions.

## Materials and methods

A qualitative methodology was applied through a content analysis of the SH protocols, codes of conduct and regulations of the seven participating European universities, between January and September 2024*. The content analysis included a total of ten protocols. To ensure clarity and consistency, the terms “code of conduct” and “regulations” were unified under the term “protocol” throughout the manuscript.

* *Changes might have been made to the analysed protocols/ regulations after this data collection.*

### University of Alicante (UA, Spain)

UA policies include a protocol to prevent and intervene in cases of SH, gender-based harassment, and other forms of discrimination and male violence. The protocol establishes clear procedures to act with full guarantees, providing appropriate psychological and legal support measures and tools for survivors. It applies to all members of the university community and reflects institution’s commitment to prevent and address gender-based discrimination and violence on campus. UA’s protocol was approved in 2015, and revised in 2019 to reflect legislative changes and align with the university’s organizational structure. It complies with Spain’s legal framework, aligning with the Spanish Constitution and key legislation like Organic Law 3/2007 on gender equality and Organic Law 1/2004 on comprehensive protection against gender violence. On July 27, 2023, the UA Governing Council approved a revised version of the protocol (BOUA-28/07/23) to better align with the university’s Coexistence Regulations and address specific issues.

### University of Palermo (UNIPA – CESIE ETS, Italy)

The UNIPA implemented the “*Code of Conduct for the Prevention of Violence, Harassment, and Discrimination in the University Context”*, in March of 2022. This document underscores UNIPA’s commitment to preventing and addressing SH, GBV, and discrimination within the academic community. The protocol outlines both formal and informal mechanisms for handling cases of SH and emphasises the need for clear definitions, awareness campaigns, and specialised training for university staff. Additionally, UNIPA aims to strengthen collaboration with local organisations and institutions (e.g., anti-violence support services, legal assistance organizations) to enhance prevention and response efforts. Future improvements include structuring survivor support services, refining case follow-up procedures for survivors and perpetrators, and ensuring comprehensive training to all the university community to foster a safer and more inclusive university environment.

### University of Maia (UMAIA, Portugal)

UMaia is firmly committed to preventing and combating SH, revealing zero tolerance for any violation of one’s dignity and dignified work. Since November 2022, the *“Código de Boa Conduta para a Prevenção e Combate à Violência e o Assédio (The Code of Good Conduct for Preventing and Combating Violence and Harassment)”* has been in place, covering different forms of discrimination and violence, including sexual and moral harassment. The Code was designed in compliance with Portuguese Labour Work Law no. 7/2009 of February 12^th^, altered by the Law no. 73/2017 of August 16^th^, which reinforces the prevention of harassment in the workplace. The protocol applies to non-teaching and teaching staff and students, in compliance with Law no. 61/2023 of November 9^th^, which extends the scope of application to all members of the academic community, including scholarship holders, interns and service providers/suppliers.

### Adam Mickiewicz University in Poznań (AMU)

AMU has implemented the *“Polityka równościowa i antydyskryminacyjna Uniwersytetu im. Adama Mickiewicza w Poznaniu (Equality and Anti-discrimination policy),”* aimed at ensuring an academic environment free from discrimination, unequal treatment, and all forms of violence, including mobbing and harassment. This document, adopted on June 6, 2022, as an annex to Order No. 232/2021/2022 of the Rector of AMU, reflects the university’s commitment to upholding the principles of equality and respect for all members of the academic community. AMU’s Equality Policy is in line with the national legal framework, including the Law on Higher Education and Science, as well as EU anti-discrimination directives. It is based on constitutional and international standards for the protection of human rights, including the Universal Declaration of Human Rights, the Charter of Fundamental Rights of the European Union, and the Constitution of the Republic of Poland. The protocol’s objective is to maintain the quality of education and ensure a safe and violence-free work and study environment, free from discrimination. Actions in this regard are to be regularly monitored and evaluated to ensure continuous improvement.

### University of Applied Sciences Burgenland (UASB, Austria)

At UASB there is no protocol for a formal report of SH. Within the scope of the Uni4Equity-project a proposal considering the existing framework conditions and structures has been developed. It has already been submitted and was presented to the management in February 2025, but further clarification is still needed regarding the procedure, access to the protocol, and the framework for exchange between the contact persons. Currently there are two documents addressing SH at the university: *“Unterstützung bei sexueller Belästigung (Support in Case of Sexual Harassment)*” and *“Richtiges Verhalten im Anlassfall bei sexueller Belästigung (Proper Conduct in Cases of Sexual Harassment)”.* They both provide an overview of the definition of SH, considering legal framework conditions, appropriate behavior in physically close teaching situations, internal and external contact points, as well as preventive measures. They will be integrated into one document *“Guideline Sexual Harassment”*.

### University of Verona (UNIVR, Italy)

The UNIVR has implemented measures to prevent and intervene in SH and other forms of discrimination. The most important of these were *“The Code of Ethics”* and *“The Regulation on Bullying and Sexual Harassment”. “The Code of Ethics”* was issued by Rectoral Decree no. 145 (10 January 2018) and contains general principles and values which UNIVR considers fundamental. Procedures are integrated by regulations issued by Rector’s decree no. 7289 of 27 July 2023. They comply with legal framework, including the Italian Constitution and key laws such as Law no.4, 2021, regarding the ratification and implementation of International Labour Organisation Convention No. 190 on the Elimination of Violence and Harassment in the Workplace, and Legislative Decree no. 198–2006 and *“The equal opportunities code opportunities between men and women*”, pursuant to Article 6 of Law no. 246–2005.

### University of Antwerp (UANTWERP, Belgium)

In December of 2023, the Flemish government enacted a decree addressing harassment in higher education. The decree requires institutions to implement a protocol on harassment, designate a central point of contact for survivors, and maintain a digital registry of all harassment reports [23]. This new regulation compels Flemish universities to critically review their existing policies and procedures. At UANTWERP, SH is formally recognised as a psychosocial risk and is addressed within the framework of the procedure for psychosocial risks at work, in line with the national Well-being Law. Thus, the UANTWERP policies include two separate documents called “*Internal procedures for harassment”* for addressing harassment when experienced by students or staff. These procedures prioritise resolving issues internally and at the closest possible level, and are embedded within UANTWERP’s broader code of conduct, which emphasises diversity and fosters a positive working and learning environment.

### Ethical considerations

Ethical approval was obtained by the research ethics committees of the participating universities (Ref. no. UA-2023-03-27; UNIPA_158/2023; UMAIA_ 151/2023; UNIVR-24/2023; UAM_19/2022/2023; UASB _28/08/2023; UANTWERP_EX_SHW_2023_38_1). This study involved a qualitative content analysis of public and available documents and materials. No human participants or biological samples were used. Therefore, no written or verbal consent was required.

### Procedures

First, the UA developed homogenized templates with common analytical categories for the data collection, including **key components** *(Name of the protocol or similar, Link to the document, Material scope, Profiles of targets of SH and perpetrators, Scope of applicability, Intersectional approach*, *Primary preventive actions, Reparation strategies, Follow-up and reparation measures for survivors and perpetrators, Coordination mechanisms between the university and external resources)* and **recommendations** *(related to each university´s SH protocol; the multisectoral response to cases of SH in the university setting* [coordination within internal and external resources], *the improvement of the follow-up of survivors and perpetrators as well as their integral care*, *the intersectional approach of the measures included in the protocol*). Next, a qualitative synthesis was conducted to identify the key components for inclusion in an SH protocol, based on recommendations from the following guidelines:

(1) The UniSAFE guideline “*Developing a Protocol for addressing gender-based violence in research and higher education institutions: UniSAFE guidelines*” [17], whose aim is to provide guidance to research and higher education institutions in designing a protocol to address GBV. This guideline explains what a protocol is and what elements it should cover and provides along with practical tips and sample practices.
(2) The “*Protocol university guideline to prevent and redress with due diligence situations of gender-based violence, sexual harassment* *and harassment based on sex, sexual orientation, gender identity or gender expression”* of the Government of Catalonia (Generalitat de Catalunya), Spain [24]. This document provides standards and recommendations to help institution design and implement prevention and reparation interventions with due diligence. It also aims to support protocol revisions by establishing a common system for collecting quantitative and qualitative indicators to monitor and evaluate protocols at the university level.

The guidelines described aim to support staff members responsible for developing and implementing SH and GBV protocols in their institutions. Each guideline provides the indicators that SH and GBV protocols should address, sharing most of them.

A first draft of the qualitative synthesis report was shared among the project university partners for major revision and contributions. After identifying the presence or absence of key components in the analysed protocols, all partners provided recommendations based on the needs found in their SH protocols.

### Key components of SH protocols in the university context

This section provides a brief description of the key components identified through the review of the two guidelines – international (UniSAFE) and national (Spain)- in order to implement SH and GBV protocols in universities. Table 1 presents an overview of the key components and Tables 2–5 further elaborate on their content.

**Table 1:** Key components of SH protocols in the university context according to the guidelines analysed^a^.

KEY COMPONENTS									
**MAIN INFORMATION**		Material Scope	Profiles of targets of SH and perpetrators	Scope of applicability	Intersectional approach				
**PREVENTIVE ACTIONS**		Information and awareness-raising campaigns	Implementation of training programs	Assessment of the working and study environment	Dissemination of the protocol (Offline/ Online)	Dissemination of the protocol (focus on minority groups)	Identification and monitoring of SH	FAQ^b^ section	Violet dots
**REPARATION STRATEGIES**	**INFORMATION AND COUNSELLING**	Inform (steps, terms, deadlines)	Guarantee of confidentiality	Offer translation services	Offer psychological support	Offer legal guidance	Offer information and different options for reporting		
	**INVESTIGATION**	Detail the investigation procedure	Hearing the parties (Interview)	Preliminary technical report	Request the procedure file	Refer the case to the competent body	Request a suspension of the procedure	Request the opening of a disciplinary proceeding	
	**SANCTION AND RECONCILIATION MEASURES**	Sanctions for staff	Sanctions for students	Educational measures	Reconciliation measures				
	**FOLLOW UP AND REPARATION ACTIONS**	Follow up of the case	Monitoring and evaluation of the protocol	Information and referral to specialised services	Protective measures	Compensatory measures	Symbolic reparation measures	Others	Access to therapeutic/ re-educational resources
**COORDINATION MECHANISMS**		Establish institutional agreements	Information about available support services						

^a^Madesi V, Polykarpou P, Mergaert L, Wuiame N. Developing a protocol for addressing gender-based violence in research and higher education institutions [Internet]. Antwerp: Yellow Window; 2023 [cited 2025 May 20]. Available from: https://unisafe-toolkit.eu/home/; Generalitat de Catalunya. Protocol guia d’àmbit universitari per prevenir i reparar ambdiligència deguda les situacions de violència masclista, assetjamentsexual i assetjament per raó de sexe, orientació sexual, identitat degènere o expressió de gènere. Generalitat de Catalunya. Generalitat de Catalunya, 2023. Available from: https://igualtat.gencat.cat/ca/inici

^b^**FAQ**= Frequently Asked Questions.

**Table 2:** Description of key components: Main information.

DESCRIPTION OF KEY COMPONENTS	
**MAIN INFORMATION**	Core elements that define the scope and applicability of the protocol, including which behaviours are considered unacceptable and sanctionable, who may be affected or involved, where the protocol applies and whether the intersectional approach underlies its implementation.
**Material Scope**	Clear definition and establishment of which behaviours are unacceptable and can be sanctioned.
**Profiles of targets of SH and perpetrators**	Reference to the profile of people who may be a target of SH or a perpetrator of SH. The protocol should assume that any member of the university community (students, faculty members and staff, internal or external such as service providers, suppliers or event participants), whether female/male or intersex, can be a potential target of SH or a perpetrator of SH.
**Scope of applicability**	Range or extent to which a particular policy, rule, procedure, or concept is relevant or applicable. It defines the boundaries within which the guidelines, requirements, or conditions apply, specifying who, what, where, or under what circumstances something is considered valid or enforceable (students, teaching and non-teaching staff, internal or external, covering behaviours both inside and outside the institution, online and offline).
**Intersectional approach**	Understanding of stereotypes and gender relations, their roots and consequences in the application and evaluation of the protocol´s impact. Ensures the protection and recognition of the rights of individuals who suffer multiple forms of discrimination or structural inequality (e.g., racialised people, people with disabilities, etc.).

**Table 3:** Description of key components: Preventive actions.

DESCRIPTION OF KEY COMPONENTS	
**PREVENTIVE ACTIONS**	Actions aimed at avoiding or reducing the incidence of the problem through the reduction of risk factors, the prevention of normalisation and raising awareness among the university community.
**Information and Awareness-raising campaigns**	Actions designed to educate the public, enhance understanding, dispel misconceptions, promote action and increase knowledge about a specific issue, cause or topic. For example, drafting informational documents on the University’s commitment, designing campaigns on the rights and freedoms of LGTBI people, and establishing awareness campaigns on the prevention of digital SV.
**Implementation of training programs**	Implementation of structured learning initiatives aimed at developing specific skills, knowledge, or competencies within a group or organisation. Such initiatives might include, for example, training with a focus on the prevention of harassment, the promotion of gender equality, sexual diversity, non-discrimination and on the prevention, awareness and detection of behaviors included in the protocol.
**Assessment of the working and study environment**	Study of the academic and work climate, the incidence and characteristics of SH behaviors and their associated risk factors, with special attention to intersectionality. The assessment will also serve to design identifiers of the problem and know the impact of SH on the health of the university community (absenteeism, decreased productivity, mobility, etc.).
**Dissemination of the protocol (face-to-face/ Online)**	Method of disseminating the protocol´s contents to a target group (the university community). This entails explaining the rules, procedures and scope of the protocol to ensure people are educated and aware of contents. Dissemination can take place through different formats, face-to-face or online.
**Dissemination of the protocol (needs of minority groups)**	Distribution of the information contained in the protocol in a clear and simple way, including adaptation of its contents to consider the needs of the social minority groups (people with disability, exchange students, etc.).
**Identification and monitoring of SH**	Improve the university’s existing procedures, tools and systems for the identification, monitoring and evaluation of incidents of SH. This involves creating mechanisms and refining processes to facilitate incident reporting, collect information and ensure follow-up of cases.
**FAQ**^**a**^ **section**	A dedicated section of a website that addresses the most frequently asked questions or concerns about a specific service or topic. A FAQ section contains clear and understandable answers to the most frequently asked questions on topics related to SH, GBV and the university´s protocol, complaints, procedures, deadlines, technical problems, etc.
**Violet dots**	Violet dots represent physical or virtual spaces that provide support, information, and assistance on GBV in any of its forms to all members of the university community.

^a^**FAQ** = Frequently Asked Questions.

**Table 4:** Description of key components: Reparation strategies.

DESCRIPTION OF KEY COMPONENTS	
**REPARATION STRATEGIES**	Reparation refers to measures taken by responsible bodies to address all areas of harm and provide necessary counselling to affected individuals. This includes two key goals: (1) reparation (comprehensive intervention from initial detection though investigation, punishment, care, counselling, and treatment); and (2) prevention of revictimization (ensuring such violence does not recur).
**INFORMATION AND COUNSELLING**	
**Inform (steps, terms, deadlines)**	Provision of information on the different steps involved in activating a protocol, including the terms established, deadlines and if certain information will be made public at the end of the process (e.g., whether or not the accused person was found guilty).
**Guarantee of confidentiality**	A fundamental principle of any SH protocol which ensures that sensitive or private information is kept secure and not disclosed to unauthorized individuals or entities. Prevents revictimisation by ensuring that individuals are provided with choices and control over their involvement in the investigation, support services, and decision-making.
**Offer translation services**	Provision of professional assistance to ensure that people who don´t speak the vernacular language or are disabled (e.g., an auditory disability) understand the information provided.
**Offer psychological support and/ or legal guidance**	Provide survivors with all available university resources, such as specialized psychological care and legal advice, regardless of whether they choose to file a formal report.
**Offer information and different options for reporting**	Both offline (e.g., university administrative office) and online (e.g., mail, phone, complaint form).
**INVESTIGATION**	
**Detail the investigation procedure**	Process defining the particular steps and methods used to conduct an internal inquiry and to resolve claims of SH. This process usually includes how the investigation begins, the roles and responsibilities of those involved, collection and analysis of evidence, how information is documented, how interviews are conducted, how confidentiality is maintained, and how decisions are reported or communicated.
**Hearing the parties (Interview)**	The process of gathering information or testimony from the people involved in the case. This includes conducting interviews in which each party has the opportunity to present their version of events, share their views and provide relevant details.
**Preliminary technical report**	Initial document presenting initial findings, observations and analysis of the reported case. This report is typically used to inform stakeholders or decision-makers about an investigation´s progress to that they can assess the situation and plan further actions. This report determines whether it is necessary to expand an investigation and convene a commission of inquiry. Once convened, a commission may carry out the following remedial measures: • **Request the procedure file**, due to insufficient evidence of conduct warranting disciplinary action or because the case falls outside the protocol’s scope • **Refer the case to the competent body,** in accordance with the regulations in force, if the investigation committee considers that the facts reported do not fall within the scope of application of the protocol (e.g., the conduct does not constitute harassing behavior), but may be subject to sanction because they constitute a behavior that violates the dignity of the person (e.g., making a sexist comment against a female colleague). • **Request a suspension of the procedure**, because the facts that are the object of the complaint may constitute a crime. • **Request the opening of a disciplinary proceeding** against the persons responsible.
**SANCTION AND RECONCILIATION MEASURES**	
**Sanctions**	A wide variety of measures taken when initiating a disciplinary procedure. Measures can include: moral condemnation (e.g., a formal oral or written warning, a call to order), material penalties (e.g., withholding of pay, fines, a refusal to grant a particular benefit), a change of situation (e.g., temporary suspension from work, change of post or laboratory), a restorative sanction (e.g., community services, restorative/educational programmes, psychological support and probation periods), and dismissal. Specific examples of sanctions include: • **In the case of staff:** disciplinary dismissal; suspension of functions, occupation or salary; forced transfer; demerit or career penalization (e.g., promotion or voluntary mobility); and warning, among others. • **In the case of students:** expulsion from the university for a period of two months to five years; loss of partial enrollment rights for an academic year or semester; prohibition of services/facilities use or pursuit of educational or learning activities; temporary (partial or complete) suspension; prohibition of participation in exams or educational activities; and warning, among others.
**Educational measures**	Measures that include participation or collaboration in training, cultural and sports activities, public health initiatives, university outreach programmes, institutional relations and more. These measures involve the agreement of both parties, and measures aim to support the reparation of the survivor, as well as the offenders´ recognition of responsibility, thus restoring the relationship between the affected parties.
**Reconciliation measures**	Measures that activate mechanisms based on dialogue (e.g., mediation).
**FOLLOW UP AND REPARATION ACTIONS**	
**Follow up of the case**	Ongoing monitoring of a situation and investigation of SH to ensure progress, gather additional information, and evaluate the process. This includes following up with the affected person, the testimonies, and support network to detect potential secondary victimization.
**Monitoring and evaluation of the protocol**	Systematic process of tracking and assessing the implementation and effectiveness of a protocol or set of guidelines to address SH. This includes monitoring compliance with established procedures, evaluating outcomes and impacts, identifying strengths and areas for improvement, and using results to inform updates or adjustments to the protocol.
**Information and referral to specialised services**	Inform and, when appropriate, referrer to internal or public services or specialised entities that provide psychological support and legal advice or other resources.
**Protective measures**	Measures implemented to protect the survivor within the university facilities through, for example, use of security services (e.g., in cases where protection or a restraining order is in force).
**Compensatory measures**	Measures that grant a specific group certain rights to redress a situation of vulnerability with respect to other groups, including specific measures for students and employees: • **Students:** Support adaptations in classes (e.g., flexibility in class attendance); Adaptations of the format of assessment or exams (e.g., additional time for the delivery of work and assessable activities); Repetition of course subjects; Administrative adaptations (e.g., change of group, timetable, seminar or tutorials); Modification of enrolment. • **Staff:** The right to a reduction or reorganisation of working time, to geographical mobility, a change of work centre, job adaptation and to the support needed due to a disability; Absences and lack of punctuality at work due to the physical or psychological impacts of a situation; and other forms of symbolic reparation as deemed appropriate.
**Symbolic reparation measures**	Measures adopted in consultation with the affected person, which may substitute or complement sanctions, considering the impact of the incident (e.g., organizing awareness campaigns against such violence, recognizing survivors publicly such as making tributes and commemorations).
**Others**	Other types of support, such as a specific fund to prevent survivors from abandoning their university studies or being affected economically.
**Access to therapeutic/ re-educational resources**	Availability of different tools, services or programs (internal or public) designed for individuals who have committed SH behaviours addressed by the protocol. These resources aim to support the development of skills, knowledge and abilities, with the goal of avoiding recurring violence.

**Table 5:** Description of key components: Coordination mechanisms.

DESCRIPTION OF KEY COMPONENTS	
**COORDINATION MECHANISMS**	Mechanisms that establish institutional agreements to ensure the protection, support, and redress of survivors of SH. These mechanisms ensure that affected individuals and communities receive timely guidance, assistance, and a coordinated response across different areas of the institution.
**Establish institutional agreements**	Agreements and collaborations with internal (e.g., university psychological care service) and external institutions (e.g., associations related to SV and/or SH, women´s centre, etc.) to define responsibilities, protocols, and procedures for handling cases, and to provide support services with all its relevant information (e.g., contacts, schedules, objectives, and locations).
**Information about available support services**	Clear and detailed guidance provided to individuals or communities regarding resources and services (external and internal) available to them in addressing SH and GBV.

## Results

### Analysis of key components of SH protocols in seven European universities

The participating European universities with a specific protocol on how incidents of SH are reported, addressed and resolved include UA, UNIPA, UMAIA, AMU and UANTWERP.

The UNIVR has two complementary protocols that address SH. *“The Code of Ethics”* covers general procedures and sanctions for investigating and dealing with breaches and includes a specific article on bullying and SH. *“The Code of Ethics”* specifies that in such cases the rector will forward the complaint directly to a confidential advisor, who will follow the procedures outlined in the corresponding regulation, in this case *“The Regulation on Bullying and Sexual Harassment”*, which describes two separate procedures (informal and formal) that the Confidential Adviser may pursue, depending on the specific situation and with the consent of the survivor.

UASB does not have a clear protocol that addresses SH. However, the university has a policy document that describes provision of support in the cases of SH and procedures for on how to manage these situations.

#### Main information.

Material scope: All of the protocols analysed address SH behaviours (UA, UNIPA, UMAIA, AMU, UASB, UNIVR, UANTWERP), as well as other discriminatory behaviors including harassment based on sex, direct and indirect discrimination based on sex (UA) and GBV (UA, UNIPA), direct (UA, AMU), indirect discrimination (UA, UNIPA, AMU, UNIVR, UANTWERP) and other forms of discrimination such as by association (practices against someone who doesn’t have a legally protected characteristic but is linked to a discriminated group) and by assumption (discrimination based on the mistaken belief that someone has a legally protected characteristic) (AMU). Some universities’ protocols also mention moral harassment (UMAIA), unequal treatment, mobbing, hate speech, bias-motivated violence and prejudge (AMU), physical/verbal aggression (UASB), power abuse, racism, and bullying (UNIVR, UANTWERP).

Profiles of targets of SH and perpetrators: All analysed documents recognize that any person in the academic community whether a student (at all levels – grade, postgraduate, doctoral, interchange students…) or a staff (research staff, teaching staff, administrative and service staff and third parties who interact with staff), can potentially be targets or perpetrators of SH (or other types of violence).

Scope of applicability: The protocols analysed protect any person belonging to the university community. This includes students and staff across different levels, roles, and hierarchical positions, and a wide range of academic, administrative, and institutional professionals (e.g., university management, people involved in training courses, research programs and academic collaborations and those interacting with local communities or attending conferences, etc.).

The protocols of UA and UMAIA, also clarify that they apply to instances of SH regardless of when or where they occur, as long as they directly impact university activities (e.g., the work and/or academic activities of members of the academic community). SH may take place during or outside of normal working hours, on or off campus, and either in person or online.

Intersectional approach: None of the protocols analysed explicitly mention intersectionality. However, in UA and UMAIA the protocols refer briefly to the rights of people with disabilities and explicitly reject all forms of discrimination. In addition, the UASB protocol makes reference to power dynamics that can increase the probability of SH victimisation and perpetration.

Table 6 provides a graphical representation of whether universities include or lack the key components regarding the main information.

**Table 6:** Main information: Key components of European SH protocols.

MAIN INFORMATION^a^	Material scope	Profiles of targets of SH and perpetrators	Scope of applicability	Intersectional approach
**University of Alicante**	X	X	X	X
**University of Palermo**	X	X	X	
**University of Maia**	X	X	X	X
**Adam Mickiewicz University**	X	X	X	
**University of Applied Sciences Burgenland**	X	X	X	
**University of Verona**	X	X	X	
**University of Antwerp**	X	X	X	

^a^The crosses (X) indicate the presence of the specific component for each university.

#### Preventive actions.

All participating universities’ policies provide information and promote awareness-raising campaigns to prevent SH, especially on significant dates such as Women’s Day (8 March) or the International Day for the Elimination of Violence against Women (25 November). The implementation of training programs is being conducted in most studied protocols (UA, UNIPA, UMAIA, UNIVR), except for AMU and UASB which do not have any workshops that focus on SH, GBV and the promotion of gender equality and non-discrimination. UANTWERP provides a training program related to bystander intervention, however, it is only aimed at staff members and PhD students. In some universities (UA, UNIPA, UMAIA, UASB, UNIVR), these preventive measures are formally established within the protocol, while in others (AMU, UANTWERP), similar initiatives are carried out at the institutional level but are not explicitly codified in the analysed SH protocols.

The prevalence of SH in the working and study environment is being assessed only in UA, UNIPA, AMU and UNIVR, through online surveys. Among the studied universities, dissemination of SH protocols takes place offline through reception programs or hiring processes (UA, UNIPA, UMAIA, AMU) and online, through institutional webpages (UA, UNIPA, UNIVR, UANTWERP), on screens in campus buildings (UANTWERP), the equality unit website and university social networks (UA). Likewise, UA’s protocol provides for sharing key information in user-friendly formats and in different languages. Both UNIPA and AMU are improving their systems for identifying and monitoring discrimination and unequal treatment, by developing mechanisms to facilitate case reporting. In this sense, the UA provides information channels and reporting forms (email, paper forms) that are visible, accessible and available on UA’s equality unit website, on its virtual campus and in physical spaces (leaflet, posters, etc.). Only UA has a Frequently Asked Questions (FAQ) section in its equality unit website that provides information on the protocol and multiple violet dots across the university campus (Table 7).

**Table 7:** Key components of European SH protocols: Prevention actions.

PREVENTION ACTIONS^a^	Information and Awareness-raising campaigns	Implementation of training programs	Assessment of the working and study environment	Dissemination of the protocol (face-to-face)	Dissemination of the protocol (Online)	Dissemination of the protocol (needs of minority groups)	Identification and monitoring of SH	FAQ^b^ section	Violet dots
**University of Alicante**	X	X	X	X	X	X	X	X	X
**University of Palermo**	X	X	X	X	X	X	X		
**University of Maia**	X	X		X					
**Adam Mickiewicz University**	X		X	X			X		
**University of Applied Sciences Burgenland**	X								
**University of Verona**	X	X	X		X				
**University of Antwerp**	X	X			X				

^a^The crosses (X) indicate the presence of the specific component for each university.

^b^**FAQ** = Frequently Asked Questions.

#### Reparation strategies.

Procedures of SH management differ across the universities, in terms of steps, reparation measures, services and organisms involved. Reparation strategies include information and counselling, investigation and follow up, and other actions related to the reparation of survivors and perpetrators.

Information and counselling: Only UA, UNIPA and AMU provide information on the different procedural steps involved in activating their protocols, the terms established and deadlines. All of the universities’ protocols focus on ensuring confidentiality, since this is a fundamental principle in addressing SH. Psychological care (UA, UNIPA, UMAIA, AMU, UNIVR, UANTWERP) and legal guidance (UA, UNIPA, AMU) are offered by some universities, as well as different reporting options – both online and in person – (UA, UNIPA, UMAIA, AMU, UNIVR, UANTWERP). However, no translation services are offered by any university for survivors who do not speak the local language (Table 8).

**Table 8:** Key components of European SH protocols: Reparation actions: Information and counselling.

INFORMATION AND COUNSELLING^a^	Inform (steps, terms, deadlines)	Guarantee of confidentiality	Offer translation services	Offer psychological support	Offer legal guidance	Offer information and different options for reporting
**University of Alicante**	X	X		X	X	X
**University of Palermo**	X	X		X	X	X
**University of Maia**		X		X		X
**Adam Mickiewicz University**	X	X		X	X	X
**University of Applied Sciences Burgenland**		X				
**University of Verona**		X		X		X
**University of Antwerp**		X		X		X

^a^The crosses (X) indicate the presence of the specific component for each university.

Investigation: All of the universities’ formal SH complaint processes include an initial interview for the parties involved. However, only the UA and UANTWERP prepare a preliminary technical report that compiles all relevant information. After compilation of a technical repot, the intervention of a commission of inquiry can be requested, which may implement the following measures: (a) a request for the procedure file, (UA, AMU, UNIVR, UANTWERP); (b) refer the case to another competent body, for example, the judicial system (UA, UNIPA, UMAIA, AMU, UNIVR, UANTWERP); (c) request a suspension of the procedure because the case does not correspond with the situations included in the protocol (UA, AMU), or; (d) a request to open a disciplinary proceeding (UA, UNIPA, UMAIA, AMU, UNIVR, UANTWERP) (Table 9).

**Table 9:** Key components of European SH protocols: Reparation actions: Investigation.

INVESTIGATION^a^	Detail the investigation procedure	Hearing the parties (Interview)	Preliminary technical report	Request the procedure file	Refer the case to the competent body	Request a suspension of the procedure	Request the opening of a disciplinary proceeding
**University of Alicante**	X	X	X	X	X	X	X
**University of Palermo**	X	X			X		X
**University of Maia**		X			X		X
**Adam Mickiewicz University**	X	X		X	X	X	X
**University of Applied Sciences Burgenland**		X					
**University of Verona**	X	X		X	X		X
**University of Antwerp**		X	X	X	X		X

^a^The crosses (X) indicate the presence of the specific component for each university.

Sanction and reconciliation measures: Depending on the profile of the perpetrator (student or staff), different sanctions can be applied (UA, UNIPA, UMAIA, UNIVR, UANTWERP). UMAIA also considers sanctions for scholarship holders, interns, and service providers/suppliers. Also, although most universities carry out a reconciliation process between parties by activating a reparation-oriented procedure based on dialogue (UA, UNIPA, UNIVR, AMU, UANTWERP), educational substitution measures are applied only in the cases of the UA, UNIVR and UANTWERP (Table 10).

**Table 10:** Key components of European SH protocols: Reparation actions: Sanction and reconciliation measures.

SANCTION AND RECONCILIATION MEASURES^a^	Sanctions for staff	Sanctions for students	Educational measures	Reconciliation measures
**University of Alicante**	X	X	X	X
**University of Palermo**	X	X		X
**University of Maia**	X	X		
**Adam Mickiewicz University**				X
**University of Applied Sciences Burgenland**				
**University of Verona**	X	X	X	X
**University of Antwerp**	X	X	X	X

^a^The crosses (X) indicate the presence of the specific component for each university.

Follow-up and reparation actions for survivors and perpetrators: UA, UNIPA, AMU, UNIVR and UANTWERP conduct specific, individualized follow-up plans in each case of SH. However, only the UA provides continuous evaluation and monitoring of the protocol. All universities except UASB provide measures of accompaniment and integral reparation such as information and referral to internal or external services (psychological support and legal guidance). UA and UNIVR also provide protective measures within university facilities by security services. Compensatory measures (e.g., flexibility in class/ work attendance, administrative adaptations) are provided by UA, UMAIA, AMU and UANTWERP, whilst symbolic reparation measures (e.g., commemorations) and other types of support are offered only by UA. UA, UNIVR and UANTWERP provide therapeutic and/or re-educational services to perpetrators (internal or external). These services are offered on a case-by-case basis depending on the type of SH situation and the characteristics of the perpetrator (Table 11).

**Table 11:** Key components of European SH protocols: Reparation actions: Follow-up and reparation actions for survivors and perpetrators.

FOLLOW UP AND REPARATION ACTIONS^a^	Follow up of the case	Monitoring and evaluation of the protocol	Information and referral to specialised services	Protective measures	Compensatory measures	Symbolic reparation measures	Others	Access to therapeutic and/or re-educational resources
**University of Alicante**	X	X	X	X	X	X	X	X
**University of Palermo**	X		X					
**University of Maia**			X		X			
**Adam Mickiewicz University**	X		X		X			
**University of Applied Sciences Burgenland**								
**University of Verona**	X		X	X				X
**University of Antwerp**	X		X		X			X

^a^The crosses (X) indicate the presence of the specific component for each university.

#### Coordination mechanisms between the university and external resources.

Although not considered a formal agreement, the UA, UNIPA, UMAIA and UNIVR have a network of contacts (e.g., public entities, associations) that they contact in the case of SH or GBV, which constitute a network of institutional coordination. Only UA, UNIPA, UASB and UANTWERP information about the available support services in their protocol or on their websites (e.g., the equality unit website) (Table 12).

**Table 12:** Key components of European SH protocols: Coordination mechanisms.

COORDINATION MECHANISMS^a^	Establish institutional agreements	Information about available support services
**University of Alicante**	X	X
**University of Palermo**	X	X
**University of Maia**	X	
**Adam Mickiewicz University**		
**University of Applied Sciences Burgenland**		X
**University of Verona**	X	
**University of Antwerp**		X

^a^The crosses (X) indicate the presence of the specific component for each university.

## Discussion

This study conducted a comparative analysis of the SH protocols of seven European universities, based on two guiding frameworks developed in earlier research. It also, provided recommendations for managing SH in higher education institutions. This content analysis suggests that most European university SH protocols do not incorporate all recommended key components. Most universities provide a comprehensive protocol that describes procedural steps to follow in cases of SH and that provide preventive and reparation measures. However, others stand out for the lack of implemented measures and the need for action, having broader and more ambiguous procedures. Although the phases for addressing SH outlined in a protocol do not necessarily need to be linear [25], it is crucial to establish a pathway that encompasses all of the different procedures that may apply depending on each specific case of SH. This includes implementation, evaluation, and monitoring of the parties involved, as well as the phases and the reparation mechanisms.

Through analysing various protocols, this study identified a set of requirements that are common to SH protocols. All of the analysed protocols include SH behaviours among others forms of conflict and direct or indirect discrimination. This reflects universities ‘awareness of the different forms of abuse and violence that occur in the university context. However, developing a specific SH protocol is advisable, as it prevents information from being scattered and ensures easier access and clearer understanding [17]. A definition of what constitutes “SH” is usually provided. This is essential to avoid misunderstanding, given the difficulty of describing SH [26–28]. In addition, protocols should further define various forms of transgressive behaviour and harassment, including online SH, to ensure that both on-campus and off-campus incidents are covered (e.g., sport, academic, cultural and other events such as university parties). It is advisable to provide examples and tools that define the contexts and populations covered on university websites, to ensure a comprehensive understanding of the framework’s scope and limitations. And, although procedures, individuals involved, and legal rights and responsibilities differ depending on a person’s position, a protocol should be applicable and accessible (e.g., through the university equality website, via the institutional Moodle platform, by email, through adapted printed materials, etc.) to the entire university community, including both students and staff [29]. In this regard, an empirical study in U.S. colleges and universities (n = 995) found that while most institutions had a publicly available sexual assault policy, smaller, private and male-majority universities were less likely to provide these resources online, highlighting the importance of ensuring accessibility of SH protocols for all members of the university community [30].

The results of our analyses of university SH protocols showed a general lack of practical application of an intersectional approach. Studies have shown the importance of applying an intersectional perspective when addressing SH in universities [24,31], as SH can take diverse forms and have different consequences for different groups [15,29,32]. This approach should be integrated across all stages of the SH management process, and should include prevention measures, assessment methodologies, early detection, access to support services, reporting procedures, comprehensive care, and follow-up [5,33,34]. Examples of such actions include: (a) ensuring that protocol information and reporting channels are available in English and other relevant languages; (b) offering translation-services; (c) providing multiple accessible resources and reporting channels (e.g., a standardised complaint form, phone, email, anonymous paper-based reports, in-person/virtual meetings…); and (d) including available channels on the protocol, faculty websites, virtual campuses, and physical spaces (e.g., leaflet, posters). At the same time, it is essential to explicitly acknowledge power asymmetries and relations of dependence, as these are deeply embedded in university hierarchies and academic cultures [35]. Despite the fact that these dynamics facilitate SH, they are rarely addressed in institutional protocols, limiting the effectiveness of preventive efforts and reinforcing organisational silence around SH [5].

While prevention measures are being implemented in most universities, significant gaps persist. For example, dissemination of the protocol to the university community is often lacking, usually due to the absence of effective communication channels (e.g., websites, social media accounts). To address this, information should be provided regularly throughout the academic year and during onboarding processes, welcome weeks, in information events, across campus buildings (e.g., posters), and the university’s communication channels. Evidence from UK universities shows that the effectiveness of SH policies depends not only on their existence but also on adopting a proactive approach, preventing incidents rather than reacting after they occur [36]. Only the UA included a FAQ section and “violet dots” as prevention tools to support the university community. Including a FAQ section on institutional websites can make the information more accessible and understandable, while creating a flowchart detailing the relationships between all relevant bodies and services can clarify who will be involved in the process. “violet dots” should be established in relevant spaces (e.g., university parties, cafeterias, sporting events, beginning of classes) to facilitate information, awareness, and counselling. Online strategies via websites and social media channels should also be implemented for awareness-raising campaigns and training programs about SH or GVB issues. These platforms are ideal for spreading the principles of equality, and for disseminating information, promoting awareness, training, resources and more [37]. Moreover, the assessment of the working and study environment, as well as the efforts to improve the identification of SH, remain insufficient in many universities. This may be due to limited data on SH reporting and the difficulties in measuring the issue [38–40]. To address this, it is advisable to conduct periodic surveys of all members of the working and academic communities.

The findings of this study highlight a widespread lack of reparation strategies, particularly in universities that either lack a specific SH protocol or are still in the early stages of developing one. The review by Rodríguez-Rodríguez and Heras- González [25] on SH action protocols in public universities in Spain underscores the need to adopt measures such as survivor care and protection from an integral perspective, addressing perpetrators, enhancing case follow-up, and strengthening cooperation between key resources and stakeholders. Regarding information and counselling, most universities adopt a personalised approach by providing psychological and legal guidance to survivors. Whether through individual or group interventions (ensuring a safe space), there is a need to prioritise trauma-informed intervention and to ensure access to legal guidance that helps survivors understand their rights [41,42].

In all cases analysed in this study, the credibility of the processes is ensured by guaranteeing confidentiality. However, there is a need to foster trust in the reporting process. To this end, it is advisable to develop a system for anonymous reporting. The objective of these systems is to identify problematic areas, such as patterns within faculties or departments, and to offer the necessary counselling and support to survivors. Institutional policies must also expressly state that no legal action can proceed without a formal complaint, which requires knowing the complainant’s full name, and clearly specify whether third-party reports can initiate formal investigations [17,24].

Even though the investigation procedure does not need to follow a linear process or replicate those of other institutions [25], this analysis shows that certain critical steps are missing at this stage. For example, there is often no comprehensive technical report documenting findings and outcomes, and continuous communication with all parties, particularly with the survivor, is lacking. To address this, it is advisable to appoint a single case coordinator, preferably from an equality office or a specialist in SH/GBV, who will be responsible for updating the survivor, explaining the status of their case at each stage, and overseeing the case from start to finish. This measure will help ensure consistency and accountability.

This analysis also revealed that only some universities specify sanctions and reconciliation measures, often due to the lack of a specific SH protocol or the absence of reparative mechanisms. It is important to note that, although reconciliation measures (e.g., mediation) can be effective for certain behaviours covered in protocols (e.g., mobbing, verbal aggression), they are not recommended in cases of SH [43,44]. Universities should define and clarify the disciplinary procedures to be adopted according to the profile of the perpetrator, the degree of intentionality, negligence, damage to the public interest, repetition or recidivism, and participation [17]. Additionally, the disciplinary committee must include representatives from the different levels of the institution and relevant groups (e.g., a student representative in cases involving students), and it must explicitly state that its decisions pertain to disciplinary matters, not criminal proceedings [17,24]. Not activating the sanctioning procedure does not prevent the adoption of accompanying measures and comprehensive reparation actions.

As mentioned before, the significant lack of strategies for follow-up and reparation actions found here is in line with previous studies on SH protocols in universities [25]. Universities must address this by implementing organized systems that guarantee efficient case tracking, a thorough evaluation of the institutional response, ongoing survivor care, and rehabilitation resources for perpetrators. In particular, a strategic plan that includes a detailed record of SH reports is necessary for an efficient case tracking system. This plan should contain important details about the process, parties involved, contact methods, phases, actions taken, and resolution outcomes (e.g., dialogue, compensatory measures, referral to other resources, sanctions). For an accurate assessment of the institutional response, it is recommended to establish common effectiveness indicators to collect quantitative and qualitative data [24]. For example, in relation to equality websites or applications, specific indicators could be developed (e.g., number of accesses, time spent, services consulted, number of cases processed), together with anonymous user satisfaction surveys to evaluate clarity, utility, service quality and functionality (e.g., barriers to access services, resolution/non-resolution of issues). Including the survivor’s perspectives and involving people across the institution would help guarantee ongoing improvement of the protocol and strengthen university policies for the prevention and redress of survivors [45]. At the same time, data collection and case tracking can contribute to the publication of updated annual reports. These reports could include an analysis of anonymised data, such as case statistics (disaggregated by sex, profile, and other identity variables), institutional data (on response times, services, resources awareness), and future measures to improve the accessibility and effectiveness of the protocol.

Providing information and referrals to specialised services (psychological, legal, and others) is an essential part of ensuring timely psychological care and enabling long-term monitoring of the university’s response. However, efforts should go beyond this, focusing on ensuring that perpetrators have access to therapeutic and re-educational resources to promote rehabilitation and prevent recidivism. To create a safer and more restorative environment, the protocol should also explicitly incorporate psychological assistance for perpetrators within a restorative and preventive framework. This approach aims to contribute to institutional safety while maintaining the focus on care and protection for survivors [46,47].

Finally, regardless of whether a protocol is in place, most universities still have very limited coordination with external mechanisms. Strengthening communication and collaboration among all actors is crucial to providing specialized support to survivors [48]. To achieve this, universities should reinforce ties with external institutions through joint initiatives, formal agreements, and greater access to legal and psychological resources. Internally, a structured coordination framework, preferably mentioned in the protocol, should be established among contact persons, equality units, and centres, including regular meetings and clear communication channels. Such actions will help create a local cooperation network for information exchange, risk assessment, and protocol improvement. Moreover, they will facilitate continuous monitoring of all actors involved, ensuring ongoing improvements in case management and survivor support. To ensure visibility and accessibility, partnerships with external entities and information about the available internal and external resources (e.g., contact details, schedules, objectives, locations) should be disseminated to all university members via institutional websites and the SH protocol (e.g., at the end of the document). Importantly, it must systematically outline the roles and responsibilities of the relevant services, integrating an intersectional perspective to successfully address the needs of all involved individuals.

### Study limitations and suggestions for future research

The study has several limitations. First, there are significant differences in the context of each university regarding equality policies. This means that each institution starts from a different baseline, reflected in the diversity of protocols for addressing SH and in how strategies are developed, structured or implemented. Additionally, some of these protocols are very recent, so they have not yet had time to tell us a story. The fear of reporting, the institutional discretion, and the lack of effectiveness indicators or protocol evaluations make it difficult to know the actual number of cases resolved through this tool. This complicates, on the one hand, a fair comparison of protocols and their outcomes, and on the other hand, it increases the community’s distrust in these resources. Furthermore, some institutions combine SH protocols with those addressing other forms of harassment or discrimination, and in some cases with GBV. While SH is a manifestation of GBV, it is essential to address it through separate protocols to ensure more targeted and effective interventions. Finally, the findings of this study may have limited generalizability beyond the universities analysed, and changes to protocols since the time of analysis may affect their current applicability and relevance.

Future studies could address the limitations of this study by conducting longitudinal evaluations of protocol effectiveness, monitoring updates, and assessing their impact over time. Comparative studies across different protocols from other universities could provide insights into how contextual factors influence protocol design and implementation of measures. Additionally, research focusing on the perspectives of survivors and other stakeholders could help identify barriers, evaluate effectiveness, and guide the development of more inclusive and responsive SH protocols.

## Conclusions

This study provides a comprehensive overview of key components that should be included in an SH protocol and provides specific recommendations for enhancing and improving such protocols, based on prior evidence [17,24]. The findings reveal that, despite the progress made in recent years regarding equality policies, substantial gaps and areas for improvement persist.

On the one hand, preventive actions remain limited in certain institutions. When such actions are undertaken, they often fall outside the framework of the established protocol. These have been created as circumstantial tools for action, failing to acknowledge the existence of structural violence or address the root causes of gender-based discrimination [46]. On the other hand, while some protocols explicitly emphasise the need for an intersectional approach, their practical application tells a different reality, as they lack concrete measures to support this approach (e.g., translation services, protocol adapted to easy language, coordination with LGBTI+ institutions.). Therefore, there is a need to develop actions that respond to SH cases and promote the inclusion of vulnerable people in the university context (e.g., migrants, people with disabilities, LGBTI+).

The implementation of reparation actions is also an area for improvement. Protection and follow-up measures, as well as the provision of services, remain limited in most universities. Measures such as improving the protection of survivors from an integral perspective, paying attention to the needs of the perpetrator, and enhancing cooperation between departments and specialised external institutions are key aspects for improvement. This highlights the importance of considering SH protocols as an integrated system for prevention, care, and reparation of the harm caused. By highlighting gaps in prevention, intersectional application and reparation mechanisms, this study provides evidence-based guidance for improving SH protocols. Strengthening coordination, ensuring accessibility, and integrating follow-up, and reparation mechanisms can enhance institutional effectiveness and foster safer and more inclusive academic environments.
